# The Motivation Journey: A Grounded Theory Study on Female Cancer Survivors’ Experience of a Psychological Intervention for Quality of Life

**DOI:** 10.3390/ijerph18030950

**Published:** 2021-01-22

**Authors:** Ilaria Durosini, Lucrezia Savioni, Stefano Triberti, Paolo Guiddi, Gabriella Pravettoni

**Affiliations:** 1 Applied Research Division for Cognitive and Psychological Science, IEO, European Institute of Oncology IRCCS, 20141 Milan, Italy; lucrezia.savioni@unimi.it (L.S.); stefano.triberti@unimi.it (S.T.); paolo.guiddi@ieo.it (P.G.); gabriella.pravettoni@unimi.it (G.P.); 2 Department of Oncology and Hemato-Oncology, University of Milan, 20122 Milan, Italy

**Keywords:** motivation, cancer survivors, psychological intervention, patient engagement, quality of life, physical activity

## Abstract

Psychological interventions are proposed to cancer survivors to support their quality of life against the emotional trauma of cancer and the side effects of treatment. Psychological interventions often require patient engagement and commitment to activities that could be more or less demanding in terms of lifestyle change (e.g., psychotherapy, sports). Analyzing participant motivations (personal aims, expectations, needs) prior to participation is useful to predict their adherence to the intervention as well as final outcomes. Yet, participant motivations may evolve during the intervention because the intervention experience turns out to be meaningful and positively challenging. The present study aimed to obtain a preliminary understanding of the process of motivation change in female cancer survivors who participated in a sport-based intervention to promote quality of life by employing a grounded theory approach. Data analysis took place alongside data collection and according to the procedure of grounded theory (“open coding”, “axial coding”, and “selective coding”) in order to describe the process of motivation change during women’s participation in psychological intervention for quality of life. On 14 women interviewed, 13 reported changing their motivation to participate during the first months of involvement, mostly changing from individualistic to group-related motivations (i.e., from self-care to friendship with other participants and enriching group membership), and from physical to psychological growth (i.e., pursuing not only physical health but also self-fulfillment). The discussion explains the preliminary aspects of the motivation change process and highlights the importance to monitor motivation dynamics within psychological interventions.

## 1. Introduction

Cancer patients’ journey does not end with successful treatment. Cancer survivors have to deal with the emotional trauma of the diagnosis, lifestyle changes that affect the quality of life and social relationships, and the anxiogenic possibility that cancer may return (fear of recurrence) [1,2]. Moreover, cancer treatments have secondary effects on survivors’ health and quality of life. For example, chemotherapy, surgery, radiotherapy could cause negative physical long-term side effects (such as vomiting, nausea, and heart failure) [3] (National Institute for Health and Clinical Excellence [NICE] 2009) and other undesirable appearance-related side effects that alter their perception of body image (such as hair loss, alopecia, skin discoloration) [4,5]. Additionally, the diagnosis and the treatment of cancer may lead to major psychological effects such as depression, emotional distress, fatigue, and poor well-being that affect several areas of life (e.g., family, work) [6,7,8]. Side effects can persist for a long time and greatly reduce patients’ ability to “restart their life after cancer” [5,9]. Patients may experience difficulty returning to their everyday lives [10,11] and face specific health challenges even after treatment [12]. It is also important to maintain a gender-informed approach to the specific experience of the lived illness: women who are dealing or have dealt with cancer in the past must face specific challenges. For example, body alterations (e.g., mastectomy) generated by cancer treatments could be particularly disruptive for women in that they affect body parts more visible and connected to the expression of their own femininity (e.g., the breast); moreover, female cancer such as uterine and ovarian put directly at risk their generativity and notably influences their personal identity and “sense of womanhood” [13,14].

For these reasons, it is paramount to support cancer survivors’ quality of life, helping them to promote personal strength, interrupt the cycle of distress, and improve well-being. Over time, several psychological interventions aimed to empower the management of emotions during care (at the time of diagnosis, during treatment), promote problem-solving methods and redefine personal resources have been proposed [15,16,17]. For example, collaborative and therapeutic interventions were performed to promote a deeper understanding of patients’ illness and to improve personal well-being [18,19,20,21]. Generally, psychological interventions should be carried out at individual or group levels. In the first scenario, patient-therapist alliance is established by psychologists to improve patients’ functional strategies to deal with their emotional burden and distress [22]. For example, in counseling/individual psychological support, the psychologist evaluates patients’ level of distress and its related causes and promotes personal resources and meaning in lives through reorienting individual priorities. Instead, in group interventions, one or more psychologists treat a group of patients and the group itself is used as a resource for therapy and personal empowerment [23]. Over the years, group therapy has been recommended as a component of standard treatment for female cancer [24]. The groups enhance patients’ social support and the expression of their disease-related emotions, encouraging them to face their problems directly, strengthen their relationships with one another and find new meaning in their lives.

Additionally, other psychological interventions often require patient engagement and commitment to activities that could be more or less demanding in terms of lifestyle change, such as sports or group physical activities that lead to an improvement in personal well-being and the creation of new social bonds [25,26]. The use of mixed interventions could lead to some beneficial effects on a variety of domains related to the quality of life [27], including improved physical functioning [28,29] and cancer-related fatigue [28,30,31]. An example of a short psychological intervention for quality of life that combines physical and psychological aspects is the “Pink is Good” project, promoted by Fondazione Umberto Veronesi. In this project, women with a history of cancer voluntarily take part in a group intervention program that combines physical exercises with psychological support. During the intervention, women strengthen their bodies through a running group training program with a professional trainer and receive advice from a nutritionist. At the same time, women receive group psychological support from psychologists in which they talk about themselves and their experiences. In these psychological sessions, the participants define their personal goals, focus on their emotions and body sensations, and work with psychologists to overcome the “sense of limit” imposed by the cancer experience. Psychologists also help women to share in the group their personal achievements obtained along the “Pink is Good” project and to acquire greater awareness of their own psychological and physical resources.

The involvement of cancer survivors in this kind of psychological intervention often requires a notable intrinsic motivation. According to the theory of planned behavior [32], participation and adherence to interventions are directly predicted by individual intention, which in turn is predicted by attitude toward behavior, subjective norm, and perceived behavioral control. Additionally, many clinicians consider patients’ motivation for psychological interventions as a crucial predictor for successful treatment [33,34,35]. Generally, patient motivation facilitates better adherence, retention, and the final outcome of therapy [36,37]. Thus, analyzing participants’ internal motivation (personal aims, expectations, needs) prior to participation in the intervention is useful to predict their engagement and success in treatment [38]. Many studies on psychological interventions analyze participant motivations to take part in them, finding that the lack of motivation at the beginning of intervention can be a source of attrition [39,40]. Motivation is a broad term referring to a variety of cognitive processes that share the capacity to represent desired behavioral outcomes and to promote behavior initiation, maintenance, and fulfillment. Broadly speaking, motivation includes many constructs coming from different approaches to psychological research, such as needs, drives, objectives and goals. Motivations may be characterized by varied intensity or effectiveness in guiding behavior; for example, they could be intrinsic or extrinsic [41]. Indeed, several studies [42] have shown that intrinsic motivation is associated with greater results and a long-term change compared to extrinsic motivation. Specifically, the literature divides motivation into intrinsic, extrinsic, amotivation, and identified regulation [41]. Intrinsic motivation refers to carrying out an activity for the sole purpose of deriving pleasure and satisfaction from it for oneself. When people engage in activities that generate psychologically satisfying experiences, they experience intrinsic motivation, which is associated with a wide range of indicators of positive functioning, including engagement, learning, creativity, performance, vitality, and well-being [43,44,45,46,47]. On the other hand, extrinsic motivation belongs to a wide variety of behaviors in which the objectives of the action go beyond those inherent to the activity itself [41]; for example, one may engage in some activity to please others without having a strong personal drive. Amotivation is defined as a complete lack of intention to engage in a certain behavior, as opposed to any form of intrinsic or extrinsic motivation [48]; for example, one engages in some activity because he or she is obliged to by external forces. Finally, individuals who experience identified regulation find the behavior to be significant and important, so they pursue it voluntarily and actively, without relying on external or internal coercive forces. Identified regulation has been shown to be a strong predictor of variables such as vitality and positive affect [49] and has sometimes been shown to be an even more effective predictor of positive outcomes than intrinsic motivation [49,50]. Behavior guided by identified regulation is likely to be maintained for a longer period than less autonomous regulations, given the more internalized nature of the rewards pursued [51,52]. According to self-determination theory, these types of motivation are related differently to various types of outcomes: intrinsic motivation would be expected to be mostly associated with positive outcomes (e.g., persistence) followed by an identified regulation, which is also associated with longer maintenance of the associated behavior. Conversely, the most negative outcomes (e.g., depressive states) will result from motivation followed by external regulation [41]. Perceived positive outcomes and success attributed to personal control may lead people to foster expectations of success and positive reactions to an exercise program. This is clearly explained by the attribution theory [53] that posits that the individual attributions made to explain outcomes will influence future behavior.

Several reviews highlighted that motivations are monitored or analyzed in the context of multiple types of psychological interventions, almost in any area where activities and psychological support are implemented to promote attitudinal or behavioral changes [54,55,56,57,58,59]. However, such analyses may not appreciate that human motivation is of a dynamic nature [60]. Whether conceived as drives or impulses, declarative aims or objectives, motivation may change over time based on lived experience while one is actively engaged in an activity.

On these bases, the present study aimed at understanding and describing the process of motivation change during female cancer survivors’ participation in a psychological intervention featuring psychological support and physical activity (the “Pink is Good” project) to promote quality of life by employing a grounded theory approach.

## 2. Materials and Methods

### 2.1. Study Setting and Participants

All the in-depth interviews were conducted in an online form by two researchers (ID, LS) with experience in qualitative research methods. Participants included in this study were selected from a larger pool of women who voluntarily adhere to an Italian group intervention featuring psychological support and physical activity to promote quality of life (the “Pink is Good” project). All of them met the following inclusion criteria: (a) to be actively enrolled in the group intervention program for at least three months, (b) have a history of female cancer, (c) have at least 18 years old, and (d) speak and understand Italian. Participants were informed by the first author of this manuscript about the study, were reassured that the participation was voluntary and asked for a written and verbal informed consent. Sampling stopped when no new themes emerged from interview data, according to data saturation [61,62]. Although it is impossible to predict the sample size needed to saturate this theory, some authors stated that typical grounded theory studies included sample sizes ranging from 10 to 60 participants [63].

### 2.2. Data Collection

Ethical approval was granted by the European Institute of Oncology, IRCCS (IEO, Milan: IEO1313). The semi-structured interviews lasted on average for 30 min and were conducted online in September 2020. All the interviews were audio-recorded with participants’ permission and then transcribed verbatim. Memos were also written throughout the process to orient and support data analysis. All the interviews included demographic (e.g., age, marital status), clinical (type of tumor), and open questions with accompanying prompts and probes to elicit extended narratives in participants about their experience about the intervention, personal goals and motivations about it (Table 1). The first questions were designed to act as icebreakers to ease the participants into the interview. The suitability and effectiveness of the interview schedule were considered after each interview, and questions were adapted to follow-up on emerging concepts and themes. This iterative process also informed the sampling strategy and the evolving themes that emerged from the data.

Data collection continued until each category was saturated and no new data emerged [64,65]. We acknowledge the potential for further research to expand, develop or modify the grounded theory described in this manuscript, and recognize that our sample may not be representative of all cancer survivors or fully explanatory to all domains of psychological interventions to quality of life.

### 2.3. Data Analysis

All stages of the research process, from study design to data analysis and the resulting grounded theory, followed guidelines from a constructivist grounded theory approach [66,67,68] and data analysis was independently conducted by three researchers alongside data collection (ID, LS, ST). This allows a progressive focusing of interviews with female cancer survivors and testing of tentative hypotheses. The constructivist grounded theory allows researchers to develop a theoretical understanding of the motivation journey during psychological intervention for quality of life. In addition, we analyzed the possible positive outcomes of this motivational change in participants. The grounded theory aims at generating concepts and theory from data rather than testing hypotheses based on existing theory. In-depth individual interviews could provide a deeper understanding of the motivations under the decision to participate in psychological intervention, allowing participants to describe their experiences, insights, and the context of their experiences.

Interviews were audiotaped and transcribed verbatim. Transcripts were analyzed according to the grounded theory’s procedure [66,67,68]. In the first step, data were organized in order to facilitate the coding phases. Coding was carried out by three researchers (ID, LS, ST) following procedures outlined by Charmaz [68]. First, the authors used “open coding” in order to identify key actions and concepts between the texts and to develop labels representing their meaning. Subsequently, the researchers used “axial coding” in order to compare and grouped the codes into broader categories. As each interview was coded, some data were included in existing codes, and new codes and categories were created to accommodate emerging concepts. Constant comparison methods [66] were used at all stages of analysis to establish bounds and contexts from codes and categories. Data were compared within and between interviews. As data collection and analysis progressed, the researchers compared codes and data from the final interviews with codes and data from the early interviews to check the relevance and applicability of interpretation of the data. These connections guided “selective coding”, whereby categories were arranged to develop a conceptual model that linked to the existing literature in the field [69]. Thoughts, ideas, and interpretations of the data were recorded by memos written along with data collection and analyzed according to grounded theory principles. These helped to reflect on the data collection process and to form the emerging theory. The authors had several meetings to discuss their interpretations and insights from data, and after an iterative discussion over many weeks, a consensus on themes was reached.

## 3. Results

### 3.1. Participants

Fourteen women with a past experience of tumor agreed to participate in this study. The majority of participants had a history of breast cancer (78.6%), and all of them had undergone multiple treatments for female tumors, including surgery, chemotherapy, radiotherapy and hormone therapy. Participants’ age ranged from 36 to 62 years (*M_age_* = 50.50, *SD_age_* = 7.43) and all of them lived in Italy alone or with their partners (see Table 2 for a detailed account of the sample). The women involved in this study were previously enrolled in a psychological group intervention to promote quality of life (the “Pink is Good” project). One of the potential participants declined participation in the research.

### 3.2. The Motivation Journey

**Figure 1:**
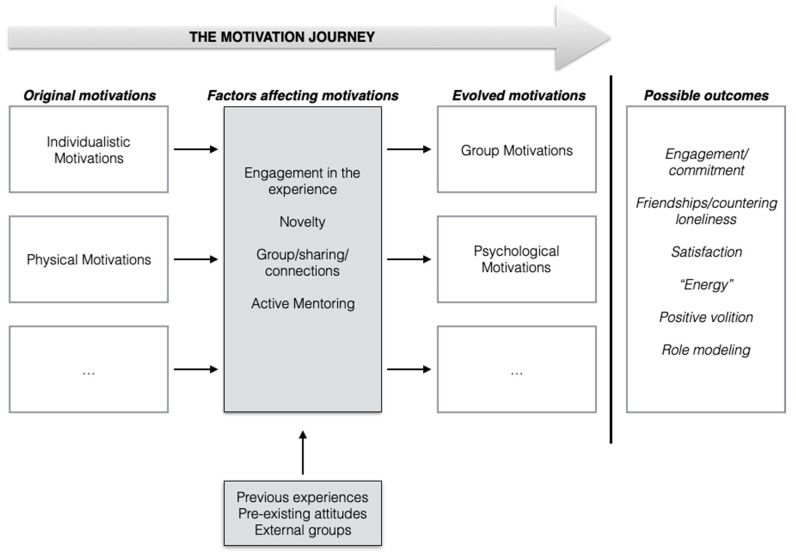
Grounded theory of how motivations change in a psychological intervention to promote quality of life (boxes with dots stand for other motivations that could possibly change in different interventions scenarios).

Among 14 women interviewed, 13 reported changing their motivation to participate in the psychological intervention during the months of involvement. These changes are characterized by different aspects and emotional dynamics that contribute to shaping the process of motivation change during female cancer survivors’ participation in the “Pink is Good” project. Our study revealed the crucial role of the group in motivation change. The interviews evidenced the evolution from individualistic to group-related motivations (i.e., friendship with other participants and enriching group membership), or from physical to psychological growth (i.e., being not only healthy but a better person too). The motivation journey was promoted by some aspects involved in the intervention. The engagement in the experience, the group sharing connections, the novelty, and the active mentoring emerged as important aspects for some participants and promoted changes in the initial motivation, and as such, is included in the grounded theory. The preliminary results of this study and the grounded theory of the process of motivation journey are presented in Figure 1, and aim to deeper understand the findings presented below. We also included in the text representative quotations of women involved in this study with hypothetical names.

#### 3.2.1. From Individualistic to Group-Related Motivations

When responding to the interview questions on initial motivations to participate in the intervention, participants reported individualistic motivations to a stronger extent. Specifically, it emerged that one of the main initial motivations was “to do something for themselves” and to “find a way to go beyond” their experience of cancer and take their life back in hand. For example, Rose (hypothetical name), a young woman with a history of breast cancer stated that:

*“I decided to join the intervention also to make sense of what had happened to me because I don’t know if I can accept the diagnosis of cancer. Surely, I learned to live with what happened to me, but I also needed to give a sense to my disease (cancer) and therefore participate in this intervention. However, (it was meant to be) also a rebirth for me; because initially it is not so simple to explain ... but there is a need to be reborn, to get cancer out of the shadows” (ID #1).*

Additionally, Elizabeth (hypothetical name) declare that:

*“I had the hope that this project would extend because it had given me an element of hope also in recovering in my normal active life; I saw it as a stage to aim for, a goal to aim for” (ID #11).*

In other cases, women started the psychological intervention featuring psychological support and physical activity with the desire to test themselves. Most of them were not athletes, but they viewed this intervention as a personal challenge and a way to overcome personal limits (e.g., improve their physical endurance) and feel *“healthier” after cancer* (*ID #9*). For example, Margaret (hypothetical name), a 54-year-old woman with a history of breast cancer, stated that:

*“For me, it is a new challenge. It is a challenge because it is a desire to achieve my personal results (...) it is not that I aspire to who knows what ... but (my personal objectives) are important to me, even just running half an hour just like I did the other day was a great satisfaction ... I feel that I would never have being able to do something like this a few years ago” (ID #8).*

Additionally, at the beginning of the intervention, participants consider this program as an opportunity to share their experience of illness. Some women explained that, in their community, stigma and shame about cancer still exist [70], and patients try to hide cancer-related feelings within social life for a long time. Women included in this program reported that their initial motivation to participate was to “take off their mask” to get rid of the burden of hiding the disease and make their voices heard. Charlotte (hypothetical name), a woman with a history of breast cancer, declared:

*“By becoming a Pink Ambassador I “come out” as a cancer survivor. After that, many people told me: “But I didn’t know (about your cancer)!” “How did you do it? Your face was always the same!”. Yes ... but it was a mask! ... and I was hiding something different. And it was nice and right because at some point (of your life) that mask has to fall down! I had been wearing it for too long. It was probably not a very conscious choice, but it was what I needed to get myself out of the situation. I thought it had to be done for me and my little daughter” (ID #7).*

After engaging in the intervention for at least three months, when responding to questions on expectations from the future and their current experience, participants reported that the experience of the group of patients was important and motivating to continue in itself. Participants underline that group-related aspects became a catalyst for their motivation to continue to take part in the intervention. Women recognized a unique *“sense of cohesion”* (*ID #12*) in the group that they never experienced in their life. For example, Elizabeth (hypothetical name) stated that:

*“I insist a lot on this aspect: for me ... the element that I have emphasized several times ... for me the team has become a really important aspect, the experience of the group … because even in moments of heated conflict, (the team) is an element of strength” (ID #11).*

Furthermore, building bonds of new social support was an important factor in maintaining motivation. Participants stated that they found new meaning in their lives by strengthening relationships with one another and feeling more energetic and “strong” in their life. For example, Margaret (hypothetical name) stated that:

*“This new group ... this feeling of being part of a group ... we feel considered in this project... (it is important) to present ourselves to others with a certain energy”(ID #8).*

Finally, it emerged that transmitting a positive message of “hope after cancer” is a motivational aspect for many of the participants. After the diagnosis of cancer, it is possible that people show a tendency to exaggerate and focus on the illness outcome and to negatively evaluate one’s ability to deal with cancer [71]. Women included in this study are strongly motivated to be a “spokesperson for life after cancer”, helping patients to “open their eyes beyond the diagnosis”. It was also very motivating for participants to spread this message within a group, making them feel “not alone” as well and useful for others. For example, Susan (hypothetical name), a 59-year-old woman declared that:

*“to make others understand that “the head matters” (the psychological aspects are important) a lot so as to believe in a possibility of normal life is a very important goal for me. (I would like to) be a spokesperson for life after cancer ... like, if you do not abandon yourself to the fatality of the tumor there is an “after”: it is not like after the disease you are a porcelain doll, like, oh my God I can’t do this I can’t do that!” (ID #9).*

In the same line, Charlotte (hypothetical name) stated that:

*“I could have been an example of how to deal with this thing even alone, while understanding that we are not alone; because now that I am “exposed”(I shared my experience) I am no longer alone, I am no longer alone because I have a group ... I am no longer alone because people know it ... I am no longer alone because I do not … anymore” (ID #7).*

#### 3.2.2. From Physical to Psychological Growth

Some patients were aware already from the start that engaging in sports within the intervention context was a “tool” to improve their psychological well-being and quality of life. They knew that the sport-focused experience was not meant to improve their strength and thinness nor their physical health only, but also to promote psychological growth. However, after engaging in the intervention, participants explicitly reported a shift of their attention and interest from the sport aspects to the psychological ones. For example, some participants highlighted, initially, a motivation to engage in sports to become healthier, physically dashing, or more thin/good-looking for personal gratification or to face upcoming treatment. Victoria (hypothetical name), a 50-year-old woman, stated that:

*“Above all, I need to tone my abdomen to do a future reconstruction surgery and the running and training they make us do will help me in this” (ID #5).*

In addition, Charlotte (hypothetical name) stated that:

*“Not necessarily to take this path of sport in my opinion, but in understanding that you can do things anyway and there are more resources that this path is giving me, resources that maybe someone can take as an example and make them their own and be motivated to also deal with the disease differently” (ID #7).*

Joanne (hypothetical name), a woman with a history of breast cancer highlighted that she wants to

*“Become aware of my body...”... “see that my body responds and that in reality I can do what I thought I could not do...” (ID #12).*

After engaging in the intervention for at least three months, the motivations changed, and respondents reported that they are now more focused on achieving personal growth than at the beginning of the intervention. From the words of some participants, it emerges the importance in finally being able to speak freely about their illness, taking off that “mask” they had been wearing for some time. This aspect has very often contributed to a personal and psychological change. For example, Emily (hypothetical name), a 55-year-old woman stated that:

*“I thought if this could be the connection that maybe makes me come back to live, to smile sincerely, not to have the mask I had put on. Because I never wanted to show others what I had inside. I always tried to have this mask, and I wanted to take it off, I wanted to be myself, joking, that never stops“ (ID #6).*

Additionally, Charlotte (hypothetical name) highlighted that:

*“Very nice for me it was the moment when I came out, declaring myself a cancer patient. How many people came to tell me they didn’t know, because my face was always the same ... yes, but it was a mask! Because that face wasn’t there behind it. And it was beautiful and right, because at a certain point that mask had to collapse, it was too long that I had worn it on me, and it was important, it was what I had needed to get me out of the situation” (ID #7).*

The renewed motivation for psychological growth appeared often tied to the testimony activity part of the project, namely the possibility for the participants to become positive ambassadors of the project and their enriching experience within external groups. Now, the motivation to be an “example” for other patients has become an important driver for participation. For example, Joanne (hypothetical name) stated that:

*“Give the opportunity to those who have lived this experience to testify that it can be done and that maybe other people (other women who are in this situation at this moment) by seeing us, they can gain strength, courage, and tell them they did. They were sick as I am sick and they did it “...” pride and pride to be able to be really helpful with my testimony to those who are going through a bad time at this moment“ (ID #12).*

Additionally, Olivia (hypothetical name), a 56-year-old woman declared that she:

*“try to sensitize more people to prevention and self-care, and to that healthy selfishness. But it’s nothing more than thinking a little more about oneself in its entirety, there is not only home, work, family, sport, but doing what makes you feel good. This is a lesson that I learned from the disease, from this experience, because I could have been gone“ (ID #4)*

At this point of the study, it was important to try to understand what factors could contribute to modify personal motivation over time. The current research did not include questions investigating this aspect specifically, especially because asking what influenced motivations directly was considered an influencing question. However, in some participants, the process of the motivation journey was clearer in that they autonomously identified characteristics of their experience that helped them to reconfigure the motivational structure orienting participation and adherence. Active involvement in the intervention (e.g., strict adherence to the activities proposed and personal initiative) was deemed important to really understand what the intervention was about and, as a consequence, to reflect on one’s own motivation beyond initial expectations and pre-existing attitudes. Similarly, a “novelty” factor in the intervention, expressed by participants’ surprise, was reported as a reason to elaborate one’s own personal aims under a new light and can lead people to feel more engaged and stimulated to renovate participation in the intervention. Finally, as clearly reported in many excerpts, social relationships within the project demonstrated an intrinsic motivational force: the experience of the positive ingroup, as well as favorable memories of the interaction with the health professionals (the psychologist and the physical trainers especially), were associated with the possibility to reframe one’s own view of the intervention as a whole, including the main drives for participation.

The changes in personal motivations helped to bring out some aspects in the participants. In particular, it was found that the evolution of the initial motivation could lead women to a greater engagement/commitment in the process. People felt more involved in all activities and requests of the intervention. Similarly, after the motivation journey, people perceived greater friendships, which countered the sense of loneliness. By changing their personal motivation, participants perceived an involvement within the group and a greater sense of closeness and support. This process also promoted satisfaction in participants, and consequently, a greater engagement in the path and a positive word of mouth. People lived the intervention with a greater physical and psychological “energy” that helped women to cope with their lives after cancer and face life with a positive volition. Finally, the motivation journey could lead women to position themselves as a “role model” for people facing cancer, sharing their experiences and thoughts. Being able to talk about their own experience and being an example for women who are now having to face cancer is often a source of pride and greater involvement in the project.

## 4. Discussion

Many studies on psychological interventions analyze participants’ motivations to take part in them. According to reviews, this is done across multiple types of psychological interventions, almost in any area where activities and psychological support are implemented to promote attitudinal or behavioral change [54,55,56,57,58,59]. Patient motivation analyzed prior to enrolment may be important to predict subsequent adherence [72,73] as well as the quality of final outcomes [74]. However, such analyses may not appreciate that human motivation is of a dynamic nature [75]. Whether conceived as drives or impulses, declarative aims or objectives, motivation could change over time while one is actively engaged in an activity. In the context of a psychological intervention for improving female cancer patients’ quality of life by organized physical activity and psychological support (“Pink is Good” project), it appeared that patients’ motivations to persist in the project activity evolved based on the ongoing experience of the intervention. In the present study, interviews data helped to preliminary reveal that changes in motivations regarded especially individuality/group (i.e., participants experienced enriching group membership within the intervention and valued sharing and others’ well-being as emerging motivational factors) and physical/psychological growth (i.e., participants had the occasion to elaborate on the sports-related activities and then expressed more personal, existential aims to achieve). Such changes in motivations are consistent with self-determination theory [41], which posits that human activity in the context of social life is implicitly directed to the fulfillment of three fundamental “nutriments”: (1) Competence, defined as feeling effective in one’s ongoing interactions within the social environment and experiencing an adequate number of opportunities to express one’s abilities; (2) Relatedness, that refers to feeling connected to others, caring and being cared, having a sense of belongingness towards individuals and community; (3) Autonomy, which relates to the perceived origin/source of one’s own behavior. Specifically, humans pursue the need of experiencing their own activity as rooted in personal interests and integrated values.

Patients in the present study lived the intervention as the opportunity to reflect on their own motivational drives so that they let emerge deep sources of quality of life and well-being as their reason to actively participate in the intervention and, more importantly, to foster their own commitment and adherence. This highlights that psychological interventions could be able to “raise” and to respond to motivations fundamental for participants’ subjective well-being and life needs, even beyond the empowerment of health management alone.

It appeared that such evolution in motivations was tied with the experience of the ongoing intervention, especially in terms of some factors that emerged from the interviews. Social aspects have an inner capacity to influence one’s goals, as the experience of a group and the possibility to share experiences with others promotes deeper reflections on one’s own motive to be there [76,77]. Furthermore, a number of patients emphasized the novelty of the overall experience, especially if compared with initial preconceptions and expectations. Psychological interventions could be unexpected and thought-provoking experiences, which help participants to explore their own goals and expectations for the future.

We argue that the “motivation journey” could affect outcomes important for any kind of psychological intervention. In other words, when evolution in initial motivations is envisaged, it could be correlated to health engagement and final satisfaction, in that participants adapt their own aims to involving experiences. Indeed, when reporting on renovated motivations, participants in the present study often referred to “a new energy” guiding their actions within the project and active participation as a whole. When patients decide to participate in a psychological intervention that is supposed to improve their quality of life, their motivations may be partial, unclear, or biased by the painful and tiring experience of illness [78]. However, it is possible that an evolution in the representation of what they could actually achieve from the intervention will influence their agency perception, promoting a positive volition or the tendency to aim for personal actualization beyond mere illness management.

As a limitation of the present study, it is important to notice that we did not implement any specific measurement of outcomes, also because the intervention analyzed here was not finished at the time of data collection. Anyway, we reported and analyzed patient testimony that, albeit at an anecdotal level, shows that participants were having a positive and enriching experience of the intervention, strictly related to a deep personal elaboration of their own motivations.

Future research should be aimed at deepening the results by studying the motivation journey of other chronic patients involved in psychological interventions. It is possible that these results may depend on the nature of the psychological intervention based on groups’ psychological support and physical activity. This point suggests the need to further investigate the motivation journey to test the transferability of this dynamic process to other interventions, as well as its possible effects on final outcomes. Additionally, a more articulated exploration of the factors affecting motivations and outcomes, especially in their dynamics, would be worthwhile in order to inform the development of methods and tools to improve participation and adherence.

## 5. Conclusions

This qualitative study aimed to obtain a preliminary understanding of the motivation journey in female cancer survivors who took part in a psychological intervention. Results of this study revealed that participants’ motivations to persist in the project activity evolved based on the ongoing experience of the psychological intervention. These changes are characterized by different aspects and emotional dynamics. Data highlighted the evolution from individualistic to group-related motivations (i.e., friendship with other participants and enriching group membership), or from physical to psychological growth (i.e., being not only healthy but a better person too). The engagement in the experience, the novelty, the group sharing connections, and the active mentoring emerged as important aspects for some participants and promoted changes in the initial motivation. This result is important for the implementation of a health intervention in that, while it is well-known that motivation influence adherence and final outcomes, it is less understood that patients’ goals and objectives may change or evolve during the intervention experience, exactly because involving activities move them to reflect on their own needs, priorities and ultimately their identity as patients or survivors. Future studies may focus on developing innovative assessment measures that take into account the complexity and dynamic nature of motivations in order to allow researchers to fully comprehend the “motivation journey” and how it affects health management and personal growth.

## Figures and Tables

**Table 1:** Interview guide.

Content Areas	Questions and Probes
Motivations/factors affecting the decision to participate in the intervention (original motivations)	*What motivated you to participate in this intervention? How did you decide to participate in this intervention? What aspects did you take into consideration to decide to participate in this intervention? (Cosa l’ha spinta a decidere di partecipare? Come ha deciso? Quali aspetti ha preso in considerazione?)* *What objectives did you set for yourself within this training experience? (Quali obiettivi si è posta con la partecipazione a questo percorso di allenamento?)* *How much do these objectives guide your motivation to train? (Quanto questi obiettivi orientano la sua motivazione ad allenarsi?)* *Are there any other factors that influenced your decision to participate in this intervention? (Ci sono altri aspetti che hanno influenzato la sua decisione di partecipare a questo programma?)*
Motivations/factor affecting engagement in the intervention after first months (evolved motivations)	*What personal benefit do you expect from participating in this intervention? (Quale vantaggio personale si aspetta dalla partecipazione al programma?)* *What are the most common feelings towards the intervention? (Quali sentimenti più comuni nei confronti del progetto?)* *How do you imagine the organization of this intervention in the coming months? (Come si immagina l’organizzazione di questa attività nei prossimi mesi?)* *What additional personal benefits do you see from participating in this intervention? (Quale ulteriore vantaggio personale vede dalla partecipazione a questo programma di allenamento?)* *What additional features should this intervention have? (Quali ulteriori caratteristiche dovrebbe avere questo programma?)*
Intervention experience and outcomes	*Have your expectations been met (to this point in time)? (Le sue aspettative sono state soddisfatte finora?)* *How was your experience until now? (Come è andata finora l’esperienza?)* *Do you want to give us any other information on your personal experience of the intervention? (Vuole darci qualche altra informazione sulla sua esperienza personale dell’intervento?)*

**Table 2:** Participant characteristics.

ID (Hypothetical Name)	Age	Education Level Reached	Marital Status	Type of Cancer Diagnosed in the Past
#1 (Rose)	36	High school	Maiden	Breast
#2 (Mary)	38	University degree or higher	Married	Breast
#3 (Catrine)	55	High school	Domestic partnership	Ovarian
#4 (Olivia)	56	Secondary degree	Married	Ovarian
#5 (Victoria)	50	University degree or higher	Married	Breast
#6 (Emily)	55	Secondary degree	Married	Breast
#7 (Charlotte)	47	University degree or higher	Married	Breast
#8 (Margaret)	54	High school	Married	Breast
#9 (Susan)	59	University degree or higher	Married	Breast
#10 (Sarah)	48	Secondary degree	Married	Uterine
#11 (Elizabeth)	46	University degree or higher	Married	Breast
#12 (Joanne)	54	University degree or higher	Maiden	Breast
#13 (Tracy)	47	High school	Maiden	Breast
#14 (Patricia)	62	High school	Married	Breast

## Data Availability

Not applicable.
